# Iron Deficiency in Acute Coronary Syndrome Treated with Percutaneous Angioplasty—A Factor of Unestablished Significance

**DOI:** 10.3390/biomedicines14051038

**Published:** 2026-05-03

**Authors:** Aleksander Misiewicz, Krzysztof Badura, Julia Wnuk-Misiewicz, Krzysztof Śliz, Maciej Nadel, Jan Krekora, Jarosław Drożdż

**Affiliations:** 1 2nd Department of Cardiology, 2nd Chair of Cardiology, Medical University of Lodz, 92-213 Lodz, Polandkrzysztof.sliz@stud.umed.lodz.pl (K.Ś.); maciej.nadel@umed.lodz.pl (M.N.); ejkrekora@op.pl (J.K.); jaroslaw.drozdz@umed.lodz.pl (J.D.); 2 Department of Internal Medicine, Asthma and Allergy, Medical University of Lodz, 90-153 Lodz, Poland; julia.wnuk@umed.lodz.pl

**Keywords:** iron deficiency, acute coronary syndrome, myocardial infarction, prognosis, anemia

## Abstract

**Background**: Iron deficiency (ID) is a prevalent condition in patients with cardiovascular diseases, irrespective of anemia, associated with adverse outcomes. Its incidence and prognostic value in acute coronary syndromes (ACS) are yet to be established. Current literature on the matter is scarce, and further research is necessary to confirm a clear link between ID and possible adverse outcome prediction in this group. Aims: This study aimed to evaluate the incidence and prognostic value of ID in ACS patients, and associations between iron parameters and patients’ characteristics, comorbidities, hospitalization length, laboratory results, electrocardiographic, echocardiographic assessment, and invasive coronary angiography results. **Methods**: We conducted an observational prospective study enrolling 214 consecutive patients after ACS. Adverse events were defined as all-cause death or non-elective rehospitalization due to cardiovascular causes. **Results**: ID patients constituted 46.7% of the studied cohort. ID was associated with higher NT-proBNP on admission (*p* = 0.03). Higher TSAT was independently associated with lower peak troponin levels (β = −0.03, standardized β = −0.15, *p* = 0.03). Ferritin < 100 ng/mL was paradoxically associated with shorter in-hospital stay (*p* = 0.03). In multivariable analysis, ID was an independent predictor of the composite endpoint (HR 1.94 [95% CI: 1.02–3.67], *p* = 0.04); however, no significant differences in event-free survival have been identified between ID and non-ID groups. **Conclusions**: ID is a common condition in ACS patients, associated with higher values of biomarkers reflecting cardiac damage, and may constitute an important predictor of adverse events after discharge. Further, larger, preferably multicenter studies are required to establish the exact association between ID and mortality among ACS patients treated with percutaneous coronary intervention.

## 1. Introduction

Iron deficiency (ID) is a mostly overlooked condition with increasing significance in cardiovascular diseases. Owing to well-established clinical trials (CTs) such as FAIR HF, IRON HF, and AFFIRM-AHF [1,2,3], the incidence and prognostic value of ID have been well studied in patients with heart failure (HF). Contrary to this, ID in acute coronary syndromes (ACS) is not a thoroughly researched condition. Although the association between ID and short-term follow-up remains ambiguous, during long-term follow-up, ID patients suffered worse outcomes [4,5,6]. ID on the cellular level is related to mitochondrial and non-oxygen-related energy production pathway dysfunction, leading to an increase in reactive oxygen species (ROS) and exacerbation of cellular damage. Moreover, regular iron serum concentration modifies the inflammatory response after AMI, presumably reducing fibrosis and adverse left ventricular (LV) remodeling [6]. To the best of our knowledge, this is the first analysis to evaluate the role of ID in ACS patients amongst the Polish population. To bridge the gap, we present the results of the study with the aim of determining the implications of ID, its incidence, and prognostic value in ACS from a single tertiary center perspective.

## 2. Materials and Methods

**Figure 1:**
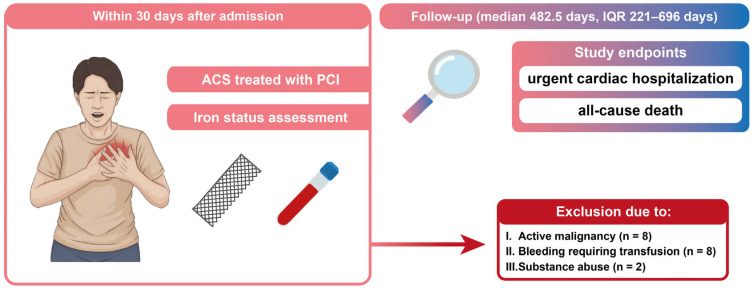
Flowchart visualizing study methodology and recruitment. ACS—acute coronary syndrome; IQR—interquartile range; PCI—percutaneous coronary intervention.

This prospective study enrolled consecutive patients hospitalized between February 2023 and August 2025 in a tertiary cardiovascular center due to ACS who were treated with percutaneous coronary intervention (PCI) with drug-eluting stent implantation. Iron parameter assessment included transferrin saturation (TSAT), ferritin concentration (FER), total iron binding capacity (TIBC), serum iron concentration (Fe), and was conducted up to 30 days after ACS in all patients regardless of their medical history. The study complied with the Declaration of Helsinki and was approved by the local medical ethics committee (RNN/298/23/KE). All patients provided written informed consent prior to their participation in the study. Exclusion criteria were as follows: severe symptomatic valve disease, active endocarditis, renal insufficiency requiring dialysis, severe liver disease, coexisting pulmonary embolism classified as intermediate-high or high risk of death, active malignancy, aortic dissection, severe anemia, bleeding within 3 months prior requiring blood transfusion, erythropoietic medicaments intake up to 6 months prior, substance abuse, pregnancy, breastfeeding, or lack of consent. The flowchart of the experiment’s methodology is shown in Figure 1. We analyzed demographic data; comorbidities; pharmacotherapy; laboratory results, including complete blood count, pro-B-type natriuretic peptide (NT-proBNP), high sensitivity cardiac troponin T (hs-cTnT) serum creatinine (sCr), estimated glomerular filtration rate (eGFR), and urea; parameters of invasive coronary angiography (lesions, stent count, amount of contrast used, radiation exposure); transthoracic echocardiography; and pre- and post-PCI electrocardiogram. Moreover, event-free survival was assessed. Events were defined as follows: all-cause death; cardiovascular non-elective rehospitalization, including visits to the emergency department, due to acute coronary syndrome, chronic coronary syndrome, angina pectoris, hypertensive emergency, acute heart failure, pulmonary embolism, and new onset of arrhythmia, including atrio-ventricular blocks (AVB), with the exception of 1st degree AVB. Data on events were obtained from the National Health Fund hospital’s database, during phone consultations with patients or their families.

ID was defined as ferritin < 100 ng/mL and/or transferrin saturation (free iron [mcg/dL]/total iron binding capacity) < 20%. Absolute ID was defined as FER < 100 ng/mL, whereas functional ID was defined as TSAT < 20% with FER ≥ 100 ng/mL. Anemia was defined as Hb < 12 g/dL in women and Hb < 13 g/dL in men [7].

Statistical analysis was performed with the use of Statistica 13.3 software (Tibco, Palo Alto, CA, USA). Tests were considered significant at a *p*-value below 0.05. Data distribution has been verified using the Shapiro–Wilk test. Nominal variables were presented as numbers and percentages or as median (interquartile range Q1–Q3 [IQR]) based on the normality of their distribution. The impact of categorical variables on ID and its subtype occurrence, as well as adverse event occurrence, was assessed using the χ^2^ test, whereas the impact of continuous variables was assessed by the U Mann–Whitney test or Kruskal–Wallis test. Significant results in multiple comparisons were calculated with Bonferroni correction. To evaluate the association of iron deficiency and iron parameters with mortality/composite endpoint, we fitted a Cox proportional hazard model that included death or urgent cardiac rehospitalization as the outcome, and all baseline variables were considered for inclusion in the multivariable models based on their relevance and their pathophysiologic significance. Association between iron status parameters and continuous variables was assessed using multivariable linear regression analysis, which included variable models based on their relevance and their pathophysiologic significance. Event-free survival was analyzed using the Kaplan–Meier method log-rank test.

## 3. Results

A total of 214 patients with diagnosed ACS and treated by PCI were included in the analysis, with a median age of 69.5 years (IQR 60.0–75.0) and a sex distribution of 64.5% male vs. 35.5% female. Median Body-Mass index was 27.9 kg/m^2^ (IQR 25.0–31.7). Full baseline group characteristics are shown in Table 1. Details of the characteristics of the ID group after division into absolute ID and functional ID are available in the Supplementary Material (Table S1).

**Figure 2:**
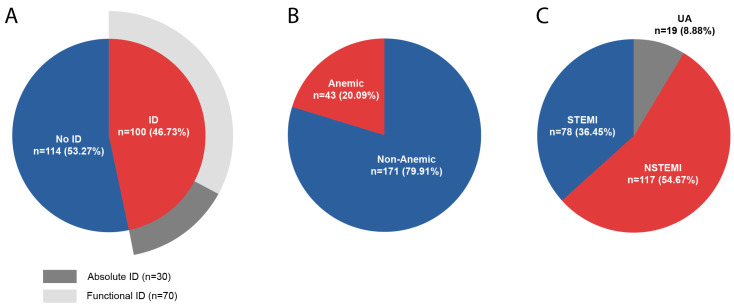
(**A**) Prevalence of iron deficiency in ACS patients. (**B**) Prevalence of anemia in ACS patients. (**C**) Subtypes of ACS in our cohort. ID—iron deficiency; NSTEMI—Non-ST-Elevation Myocardial Infarction; STEMI—ST-Elevation Myocardial Infarction; UA—unstable angina.

ID was present in 100 patients (46.7%), 86 (40.2%) had TSAT < 20%, 30 (14.0%) had FER < 100 ng/mL, and 16 fulfilled both criteria (7.5%). Absolute ID was present in 30 patients (30.0% of ID patients), whereas functional ID was present in 70 patients (70.0% of ID patients). Figure 2 presents the distribution of ID, its subtypes, anemia, and subtypes of ACS in our cohort. Hazard ratio of ID and ID subtypes in subgroup analysis is presented in Figure S1.

The median time of blood sampling was 15.0 days [IQR 12.0–18.0 days]. There was no difference in the timing of iron parameter sampling among patients with ID and without ID (median: 14 [IQR 11–19.5] vs. 15 [IQR 12–18] days, respectively, *p* = 0.40). After dividing patients into three groups (no ID, absolute ID, and functional ID), no significant differences in sampling time were observed. No differences in FER and TSAT between groups were observed in patients with early sampling and late sampling (Table S2).

### 3.1. ID and Biomarkers of Cardiac Injury and Dysfunction

**Figure 3:**
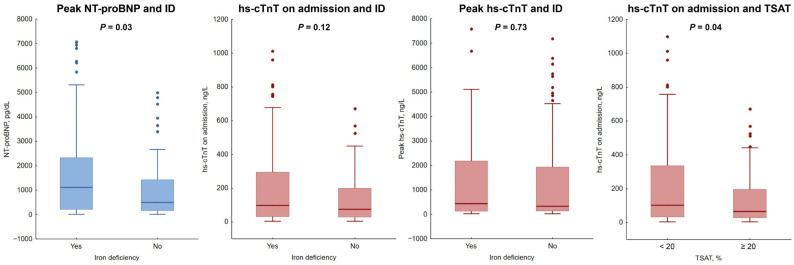
Iron deficiency and cardiac biomarkers. Associations between iron deficiency and peak NT-proBNP, hs-cTnT on admission, and peak hs-cTnT. Horizontal line within box represents median, box represents interquartile range Q1–Q3, whiskers represent range of non-deviant values, and black points represent deviant measurements. CI—confidence interval; ID—iron deficiency; hs-cTnT—high-sensitivity cardiac troponin; NT-proBNP—N-terminal pro-B-type natriuretic peptide; TSAT—transferrin saturation.

ID was associated with higher NT-proBNP on admission (median 516.0 vs. 1108.0 pg/dL [IQR 162.0–1463.0 vs. 217.3–2320.0 pg/dL] *p* = 0.03) and lower Hb (median 14.2 vs. 13.4 [IQR 13.4–15.2 vs. 12.3–14.6 g/dL] *p* = 0.001), whereas no significant association between ID and hs-cTnT was found (Figure 3). Statistically significant difference in NT-proBNP on admission was only observed in the whole group. When analyzed separately, no significance was found between the non-anemic and anemic subgroups. Lower Hb may be associated with higher NT-proBNP, due to the fact that a negative correlation between Hb and NT-proBNP (r = −0.264, *p* < 0.001) was found. Anemia was associated with higher NT-proBNP (median 538.9 vs. 1411 pg/mL [IQR 168.0–1610.0 vs. 473.6–3939.0 pg/mL] *p* = 0.003) along with lower eGFR (median 74.6 vs. 57.2 mL/min/1.73 m^2^ [IQR 58.9–92.1 vs. 41.3–81.7 mL/min/1.73 m^2^] *p* = 0.003).

After the dichotomization of TSAT into subgroups—less than 20% and greater than or equal to 20%— the first group was associated with higher hs-cTnT on admission (median 70.0 vs. 102.0 ng/L [IQR 29.0–202.0 vs. 33.0–336.0 ng/L] *p* = 0.04).

In multivariable linear regression analysis with log-transformed peak hs-cTnT as the dependent variable, higher TSAT was independently associated with lower peak troponin levels after adjustment for FER, eGFR, age, sex, prior heart failure, and LVEF (β = −0.03, standardized β = −0.15, *p* = 0.03). Lower LVEF was the strongest independent predictor of higher peak hs-cTnT (β = −0.07, standardized β = −0.44, *p* < 0.001), while older age was also associated with higher hs-cTnT (β = −0.03, standardized β = −0.20, *p* = 0.02).

### 3.2. ID and Length of In-Hospital Stay

Absolute ID (FER < 100 ng/mL) was surprisingly associated with shorter in-hospital stay when compared to patients with FER > 100 ng/mL (median 7.0 vs. 5.50 days [IQR 5.0–8.0 vs. 4.0–7.0 days], *p* = 0.03). This association was present only among non-anemic patients with FER < 100 ng/mL (median 5.0 vs. 6.5 days [IQR 4.0–6.0 vs. 5.0–8.0 days] *p* = 0.048), whereas it was not observed in the anemic group (median 5.0 vs. 8.0 days [IQR 4.0–8.0 vs. 4.0–9.0 days] *p* = 0.19). Anemia was not significantly associated with prolonged in-hospital stay when compared to non-anemic patients, irrespective of ID (median 7.5 vs. 6.0 days [IQR 4.0–9.0 vs. 5.0–8.0 days] *p* = 0.19).

### 3.3. Predictors of Adverse Cardiovascular Events and/or Death

**Figure 4:**
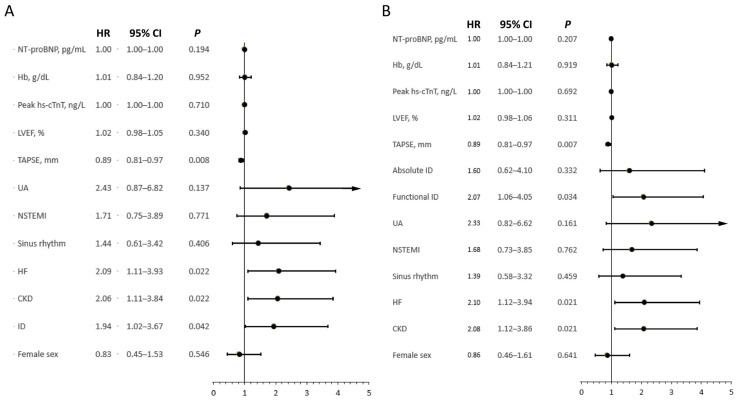
Forest plot representing effects of variables included in multivariable Cox’s proportional hazard model with undivided ID (**A**) and ID separated into subtypes (**B**). CI—confidence interval; CKD—chronic kidney disease; Hb—hemoglobin; HF—heart failure; hs-cTnT—high sensitivity cardiac troponin T; HR—hazard ratio; ID—iron deficiency; LVEF—left ventricular ejection fraction; NSTEMI—Non-ST-Elevation Myocardial Infarction; NT-proBNP—N-terminal pro-B-type natriuretic peptide; TAPSE—tricuspid annular peak systolic excursion; UA—unstable angina.

In the multivariable proportional Cox’s hazard model, ID (irrespective of absolute and functional subtype) was independently associated with increased risk of composite endpoint occurrence (HR 1.94 [95% CI: 1.02–3.67], *p* = 0.04). The results of the multivariable proportional Cox’s hazard model are presented in Figure 4. After creating a model with absolute and functional ID as separate predictors, only functional ID was independently associated with increased risk of composite endpoint occurrence (HR 2.07 [95% CI 1.06–4.05], *p* = 0.03).

**Figure 5:**
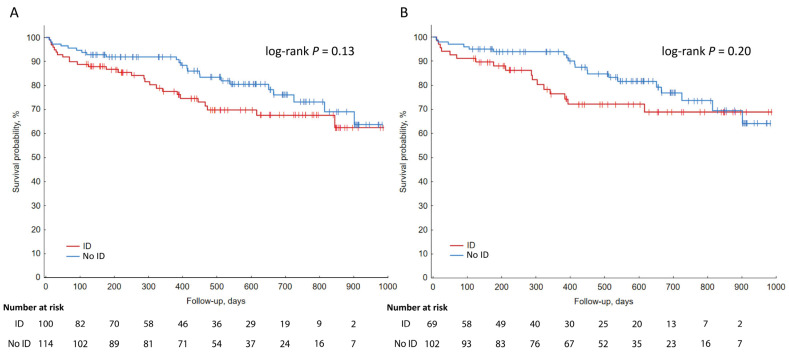
Kaplan–Meier curves illustrating post-discharge event-free survival. (**A**) Total group. (**B**) After exclusion of anemic patients. ID—iron deficiency.

**Figure 6:**
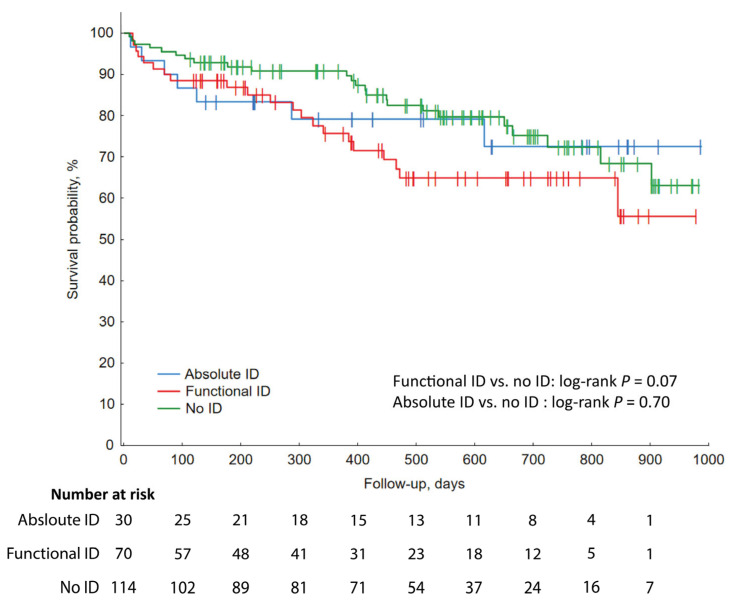
Kaplan–Meier curves illustrating post-discharge event-free survival among patients without ID and with absolute or functional ID. ID—iron deficiency.

Follow-up was available for 214 patients. Median follow-up was 482.5 days (IQR 221–696 days). During follow-up, 12 patients died, and 40 patients had non-elective cardiac rehospitalization. A breakdown of the individual causes of rehospitalization is available in Supplementary Material (Table S3). No differences in event-free survival were observed between ID and non-ID patients (Figure 5A). After the exclusion of anemic patients, follow-up was available for 171 patients. Median follow-up was 509 days (IQR 233–696 days). Similarly to total group analysis, no differences in event-free survival were observed between ID and non-ID groups (Figure 5B). After differentiating ID into subgroups—functional and absolute ID—no differences in follow-up were found (Figure 6).

## 4. Discussion

The main findings of our study are that (1) ID is a common condition amongst patients with ACS, (2) ID may be associated with volume overload on admission represented by higher NT-proBNP, (3) TSAT may be associated with an extent of myocardial damage represented by hs-cTnT, (4) low ferritin concentration could be associated with better short-term outcomes, and (5) ID is an independent predictor of composite endpoint, but in our cohort event-free survival did not differ significantly between ID and non-ID groups.

ID is a condition with growing importance as a distinct disease entity in recent years. Even in developed countries, such as the United States, 15% of adults suffer from functional and 14% from absolute iron deficiency [8]. From a European perspective, according to the Gutenberg Health Study, the prevalence of ID seems to be even higher, with functional ID accounting for 55% and absolute ID for 12% of the studied cohort. Interestingly, the incidence had risen by 19% and 5% accordingly during a 5-year follow-up [9]. As cardiovascular diseases still constitute a leading cause of death, the identification of potential modifiable risk factors seems crucial. In ACSs, the incidence and implications of ID have not been sufficiently investigated. With regard to currently available data, the prevalence of ID in ACS patients ranges from 29.1% to 62% [6,10,11] and depends on studied populations, ID definitions, timing of blood sampling, and outcomes of interest. In our study, the incidence of ID among ACS patients was 46.7%, which was comparable with currently available evidence. ID was associated with a higher concentration of NT-proBNP on admission, suggesting greater volume overload. Although iron supplementation in ID patients with HF has been shown to be highly beneficial in groups with reduced or mildly reduced ejection fraction [12], data on cardiac biomarkers and ID remain scarce. Observational studies and CTs on ID in HF alike have not shown differences in NT-proBNP depending on iron status [13,14]. In our study, a correlation between Hb and NT-proBNP has been found, with anemic patients having a significantly higher concentration of NT-proBNP. Moreover, patients with anemia had lower eGFR; however, no significant differences in eGFR have been observed among ID and non-ID patients, constituting one of the most important factors influencing NT-proBNP levels [12]. Chronic kidney disease and kidney injury can be the culprit of anemia. Erythropoietin (EPO), stimulating red blood cell production, is synthesized by periglomerular cells situated in the kidney cortex. Lower eGFR can relate to renal function impairment and, as a consequence, reduced EPO production, resulting in anemia and, simultaneously, volume overload, represented by higher NT-proBNP due to kidney filtration function impediment [15]. Further evaluation of these interactions could be assessed by urinalysis, enabling more precise chronic kidney disease diagnosis; however, in our case, the data was lacking.

Taking into consideration iron status and myocardial damage, higher TSAT was independently associated with lower peak hs-cTnT. Moreover, TSAT < 20% was associated with higher hs-cTnT on admission. A cohort study including 420 patients with STEMI has shown a greater increase in troponin among ID patients [4]. Moreover, larger infarct sizes and worse LV systolic function had been observed among ID patients [13]. From a pathophysiological point of view, decreased TSAT results in decreased iron supply to ischemic cardiomyocytes. As enzymes neutralizing reactive oxygen species (ROS) require protoporphyrin IX, having iron in its structure, it is possible that ID cardiomyocytes are more prone to ischemia–reperfusion injury due to an imbalance between reperfusion-related ROS formation and neutralization. ROS-related injury may result in greater troponin increase and larger infarct size—a phenomenon precisely described in our previous article [6].

In our study, low ferritin concentration in non-anemic patients was paradoxically associated with shorter in-hospital stay—a result which aligns with the findings of Cosentino [4] and Obradowic et al. [5] showing better short-term in-hospital outcomes in non-anemic ID patients. Ferritin is an acute phase reactant located intracellularly, the serum concentration of which is upregulated during cellular damage, when protein is released into the bloodstream [16,17]. Elevated FER could coincide with the extent of tissue damage during ischemia, resulting in myocardial injury and peri-AMI shock.

According to the results from our cohort, ID was an independent predictor of the composite endpoint of all-cause death or non-elective cardiac rehospitalization. This finding aligns with the results of Zeller et al. [18] and Silva et al. [19], which confirmed ID as an independent predictor of cardiovascular death or non-fatal MI and any-cause death or development of severe HF, respectively. In contrast to the research cited above, the ID and non-ID groups in our cohort did not differ in terms of event-free survival during mid- to long-term follow-up, irrespective of anemia (Figure 5). These results are comparable to those demonstrated by Masini et al. [11]. Studies conducted by Silva et al. [19], Zeller et al. [18], and Jenca et al. [20] showed worse post-discharge outcomes in ID patients during long-term follow-up. The reason for the possible adverse prognosis in this group is yet to be confirmed, but some causes are worth mentioning. In vivo experiments have shown a reduction in viability in cardiomyocytes subjected to hypoxic conditions and abnormally high or low iron availability in comparison to those in a hypoxic environment and with regular iron concentration [21]. Also, regular iron serum concentration could be beneficial, as macrophages, migrating to infarcted myocardium, take up iron, shifting their phenotype to an anti-inflammatory one, potentially reducing fibrosis and myocardial scarring. Based on animal models, the currently available evidence seems to be insufficient. Intravenous iron salt administration in rats previously fed an iron-deficient diet led to favorable outcomes—the reversal of anemia-induced cardiac remodeling, attenuation of fibrosis, oxidative and nitrosative stress markers [21]. Similar results were not acquired in non-ID rats subjected to ischemia and reperfusion treated with ferric carboxymaltose administration. There were no differences between groups with regard to post-MI mortality, LV remodeling, systolic function, ventricular arrhythmias, oxidative stress, and inflammation markers [22]. Iron supplementation in AMI patients has been studied in an experiment conducted by Florian et al. [23], the results of which showed improved infarct healing, beneficial LV remodeling in STEMI patients administered with an iron-rich contrast agent in comparison to controls; however, due to a lack of iron parameter assessment and the limited study group, the acquired results should be treated with caution. A currently ongoing trial, “INFERRCT” (NCT05759078, https://clinicaltrials.gov/study/NCT05759078, accessed on 17 September 2025), assessing the effect of intravenous ferric carboxymaltose on outcomes among ID patients with AMI, may provide crucial data for further investigating this relationship.

Some limitations of our study should be acknowledged. Firstly, due to the observational design, the potential for unmeasured confounding and/or selection bias cannot be ruled out. Results pertaining to prognosis should be treated with caution due to the relatively small sample size and small number of endpoints that were met during follow-up. The low statistical power of survival analysis should be emphasized. The statistical power for the log-rank test to assess the difference in survival between patients with ID and without ID was 0.21 (alpha 0.05). To reach a statistical power of 0.8, assuming a difference in composite endpoint occurrence of 7 percentage points between ID and non-ID groups, at least 593 patients in each group should have been enrolled. Moreover, the observation period was shorter than in the works cited above, which evaluated long-term prognosis. Most patients included in this study were classified as NSTEMI, with a low representation of patients with UA and STEMI. It should be noted that TSAT and FER are acute-phase reactants. Although iron parameter analysis in our research was conducted after an acute phase of ACS, when TSAT and Fer had returned to their pre-AMI values, the assessment was conducted at different time frames, potentially influencing ID diagnosis. Our study constitutes the first of its kind in Poland, conducted from a single center perspective; the results drawn here therefore need to be validated in other populations.

## 5. Conclusions

Iron deficiency is a prevalent condition in patients after ACS. Regular iron status may relate to a lesser extent of myocardial injury. Low ferritin, as an acute phase protein, could coincide with shorter in-hospital stay in this group. Both ID and functional ID in our cohort were independent predictors of composite endpoint occurrence. As for follow-up, there were no significant differences across ID and non-ID groups regarding event-free survival, even after division into absolute and functional ID groups. Acquired results should be treated with caution due to the exploratory nature and limitations of our work. Further research and multicenter approaches are necessary, especially to prove ID as a modifiable risk factor of adverse outcomes.

## Figures and Tables

**Table 1:** Baseline group characteristics. Numbers in second, third, and fourth columns represent median (interquartile range Q1–Q3) unless stated otherwise.

	Total (n = 214)	Non-ID (n = 114)	ID (n = 100)	*p*
Age, y	69.5 (60.0–75.0)	69.0 (59.0–74.0)	70 (61.0–76.0)	0.25
Male, n (%)	138 (64.49)	86 (75.4)	52 (52.0)	<0.001
BMI, kg/m^2^	27.9 (25.0–31.7)	28.41 (25.0–31.4)	27.4 (24.6–31.7)	0.80
Nicotine abuse, n (%)	128 (59.8)	66 (58.0)	62 (62.0)	0.58
Arterial hypertension, n (%)	196 (91.6)	104 (91.2)	92 (92.0)	0.97
DM, n (%)	73 (34.1)	43 (29.8)	39 (39.0)	0.19
on insulin, n (% of DM patients)	21 (28.8)	9 (22.0)	12 (30.8)	0.80
Chronic HF, n (%)	44 (20.6)	26 (22.8)	18 (18.0)	0.46
AHF on admission, n (%)	31 (14.5)	14 (12.3)	17 (17.0)	0.33
Chronic lung disease, n (%)	32 (15.9)	13 (11.4)	19 (19.0)	0.13
Stroke/TIA, n (%)	12 (5.6)	3 (2.6)	9 (9.0)	0.047
AF, n (%)	23 (10.8)	11 (9.7)	12 (12.0)	0.66
CKD, n (%)	44 (20.6)	22 (19.3)	22 (22.0)	0.67
Peptic ulcer disease	21 (9.8)	12 (10.5)	9 (9.0)	0.81
Anemia, n (%)	43 (20.0)	11 (9.7)	32 (32.0)	<0.001
Hb, g/dL	13 (12.9–14.8)	14.2 (13.4–15.2)	13.40 (12.3–14.6)	<0.001
Free iron, µmol/L	12.20 (9.1–15.6)	14 (12.4–17.3)	9.1 (7.1–11.2)	<0.001
Fer, ng/mL	230 (134.0–381.0)	273.0 (165.0–475.0)	191 (90.0–325.0)	<0.001
TIBC, µmol/L	56.72 ± 9.2	56.18 ± 8.7	57.35 ± 9.8	0.30
TSAT, %	21.00 (16.0–27.0)	27.00 (23.0–31.0)	15.00 (14.0–18.0)	<0.001
Timing of iron parameters sample collection (after ACS diagnosis), days	15 (12.0–18.0)	15 (12.0–18.0)	14 (11.0–20.0)	0.56
RBC 10^6^/mcL	4.56 (4.2–4.9)	4.58 (4.3–4.9)	4.55 (4.1–4.9)	0.21
PLT 10^3^/mcL	240 (195.0–290.0)	224 (185.0–283.0)	254 (204.0–306.5)	0.06
sCr, mmol/L	83.40 (69.3–97.5)	83.10 (71.7–95.7)	82.20 (67.0–103.0)	0.98
eGFR, ml/min/1.73 m^2^	71.3 (56.2–88.7)	74.1 (57.2–92.1)	69.8 (52.0–87.1)	0.15
NT-proBNP, pg/mL	621.0 (183.0–1896.0)	516 (162.0–1463.0)	1108 (217.3–2320.0)	0.03
hs-cTnT on admission ng/L	84 (32.0–249.0)	75 (30.0–202.0)	99.5 (32.0–292.0)	0.12
hs-cTnT peak ng/L	405 (32.0–249.0)	360.50 (143.5–1935.5)	420 (131.0–2181.0)	0.73
**Transthoracic echocardiography**				
LVEF (BiP), %	50.00 (45.0–58.0)	50.00 (45.0–58.0)	51.50 (45.0–58.0)	0.96
TAPSE, mm	21.78 ± 3.6	21.97 ± 3.6	21.57 ± 3.6	0.54
**Invasive coronary angiography and intervention during hospitalization**				
PCI, n (%)	197 (92.1)	102 (89.5)	95 (95.0)	0.22
Significant stenosis, n (%)	204 (95.3)	107 (93.9)	97 (97.0)	0.58
IRA				
LMCA, n (%)	24 (11.2)	10 (8.8)	14 (14.0)	0.27
LAD, n (%)	146 (68.2)	79 (69.3)	67 (67.0)	0.45
LCx, n (%)	89 (41.6)	52 (45.6)	37 (37.0)	0.13
RCA, n (%)	123 (57.5)	60 (52.6)	63 (63.0)	0.21
MVD, n (%)	143 (66.8)	79 (69.3)	64 (64.0)	0.41
**Number of stents implanted**				
0, n (%)	17 (7.9)	12 (11.5)	5 (5.0)	
1, n (%)	98 (45.8)	49 (43)	49 (49.0)	
2, n (%)	64 (29.9)	36 (31.6)	28 (28.0)	
3, n (%)	23 (10.8)	12 (10.5)	11 (11.0)	
4, n (%)	9 (4.2)	3 (2.6)	6 (6.0)	
5, n (%)	3 (1.4)	2 (1.8)	1 (1.0)	
Total stents implanted, n	1.00 (1.0–2.0)	1.00 (1.0–2.0)	1.00 (1.0–2.0)	0.72
Contrast media, ml	110.00 (80.0–150.0)	110.00 (80.0–150.0)	110.00 (80.0–150.0)	0.74
EFD, mGy	427.50 (256.0–686.0)	442.00 (278.0–700.0)	422.00 (238.0–671.0)	0.61

ACS—acute coronary syndrome; AF—atrial fibrillation; AHF—acute heart failure; BiP—biplane; BMI—body-to-mass index; CKD—chronic kidney disease; DM—diabetes mellitus; eGFR—estimated glomerular filtration rate; EFD—effective absorber dose of radiation; Fer—ferritin; Hb—hemoglobin; hs-cTnT—high sensitivity cardiac troponin T; ID—iron deficiency; IRA—infarct related artery; LAD—left anterior descending coronary artery; LCx—left circumflex coronary artery; LMCA—left main coronary artery; LVEF—left ventricular ejection fraction; MVD—multivessel disease; NT-proBNP—N-terminal pro-B-type natriuretic peptide; PCI—percutaneous coronary intervention; PLT—blood platelets; RBC—red blood cells; RCA—right coronary artery; sCr—serum creatinine; TAPSE—tricuspid annular peak systolic excursion; TIA—transient ischemic attack; TIBC—total iron binding capacity; TSAT—transferrin saturation.

## Data Availability

Research data can be shared with other scientists upon reasonable request.

## Supplementary Materials

The following supporting information can be downloaded at https://www.mdpi.com/article/10.3390/biomedicines14051038/s1: Table S1. Baseline characteristics with division for ID subtypes; Table S2. Differences in iron parameters in regard to early and late sampling; Table S3. Endpoints breakdown divided into groups of interest—all-cause death and causes of non-elective rehospitalization; Figure S1. Hazard ratio of ID/ID subtypes in subgroup analysis.

## Abbreviations

ACS—acute coronary syndrome; AF—atrial fibrillation; AFFIRM-AHF—Acute Heart Failure Ferric Carboxymaltose Assessment Trial; AH—arterial hypertension; AHF—acute heart failure; AMI—acute myocardial infarction; BiP—biplane; BMI—body-to-mass index; CI—confidence interval; CKD—chronic kidney disease; CRP—C-reactive protein; CT—clinical trial; DM—diabetes mellitus; eGFR—estimated glomerular filtration rate; EFD—effective absorber dose of radiation; EPO—erythropoietin; Fe—serum iron; FER—ferritin; Hb—hemoglobin; HF—heart failure; HR—heart rate; hs-cTnT—high-sensitivity cardiac troponin T; ID—iron deficiency; IRA—infarct related artery; IQR—interquartile range; IRON-HF—Intravenous Iron Therapy in Patients with Chronic Heart Failure and Iron Deficiency; LAD—left anterior descending coronary artery; LCx—left circumflex coronary artery; LMCA—left main coronary artery; LV—left ventricle; LVEF—left ventricular ejection fraction; MCHC—mean corpuscular hemoglobin concentration; MVD—multivessel disease; NSTEMI—non-ST-segment elevation myocardial infarction; NT-proBNP—N-terminal pro-B-type—natriuretic peptide; PCI—percutaneous coronary intervention; PLT—blood platelets; RBC—red blood cells; RCA—right coronary artery; ROS—reactive oxygen species; sCr—serum creatinine; STEMI—ST-segment elevation myocardial infarction; TAPSE—tricuspid annular peak systolic excursion; TIA—transient ischemic attack; TIBC—total iron binding capacity; TNT—cardiac troponin T; TSAT—transferrin saturation; UA—unstable angina.

## References

[1] Anker S.D., Comin Colet J., Filippatos G., Willenheimer R., Dickstein K., Drexler H., Lüscher T.F., Bart B., Banasiak W., Niegowska J. (2009). Ferric Carboxymaltose in Patients with Heart Failure and Iron Deficiency. N. Engl. J. Med..

[2] Beck-da-Silva L., Piardi D., Soder S., Rohde L.E., Pereira-Barretto A.C., De Albuquerque D., Bocchi E., Vilas-Boas F., Moura L.Z., Montera M.W. (2013). IRON-HF Study: A Randomized Trial to Assess the Effects of Iron in Heart Failure Patients with Anemia. Int. J. Cardiol..

[3] Ponikowski P., Kirwan B.-A., Anker S.D., McDonagh T., Dorobantu M., Drozdz J., Fabien V., Filippatos G., Göhring U.M., Keren A. (2020). Ferric Carboxymaltose for Iron Deficiency at Discharge after Acute Heart Failure: A Multicentre, Double-Blind, Randomised, Controlled Trial. Lancet.

[4] Cosentino N., Campodonico J., Pontone G., Guglielmo M., Trinei M., Sandri M.T., Riggio D., Baggiano A., Milazzo V., Moltrasio M. (2020). Iron Deficiency in Patients with ST-Segment Elevation Myocardial Infarction Undergoing Primary Percutaneous Coronary Intervention. Int. J. Cardiol..

[5] Obradovic D., Loncar G., Zeymer U., Pöss J., Feistritzer H., Freund A., Jobs A., Fuernau G., Desch S., Ceglarek U. (2024). Impact of Anaemia and Iron Deficiency on Outcomes in Cardiogenic Shock Complicating Acute Myocardial Infarction. Eur. J. Heart Fail..

[6] Misiewicz A., Badura K., Matuszewska-Brycht O., Krekora J., Drożdż J. (2025). Iron Deficiency as a Factor of Worse Prognosis in Patients with Acute Myocardial Infarction. Biomedicines.

[7] World Health Organization (2024). Guideline on Haemoglobin Cutoffs to Define Anaemia in Individuals and Populations.

[8] Auerbach M., DeLoughery T.G., Tirnauer J.S. (2025). Iron Deficiency in Adults: A Review. JAMA.

[9] Schrage B., Rübsamen N., Schulz A., Münzel T., Pfeiffer N., Wild P.S., Beutel M., Schmidtmann I., Lott R., Blankenberg S. (2020). Iron Deficiency Is a Common Disorder in General Population and Independently Predicts All-Cause Mortality: Results from the Gutenberg Health Study. Clin. Res. Cardiol..

[10] Barrabés J.A., Inserte J., Castellote L., Buera I., Milà L., Sambola A., Uribarri A., Vidal M., Aluja D., Delgado-Tomás S. (2025). Iron Deficiency Is Associated with Impaired Myocardial Reperfusion in ST-Segment–Elevation Myocardial Infarction: Influence of the Definition Used. J. Am. Heart Assoc..

[11] Masini G., Barsacchi M., Chiusolo S., Alberti M., Gargani L., Corradi F., De Caterina R. (2025). Iron Deficiency in Acute Coronary Syndromes—Clinical Correlates and Outcomes. Am. J. Med..

[12] McDonagh T.A., Metra M., Adamo M., Gardner R.S., Baumbach A., Böhm M., Burri H., Butler J., Čelutkienė J., Chioncel O. (2023). 2023 Focused Update of the 2021 ESC Guidelines for the Diagnosis and Treatment of Acute and Chronic Heart Failure. Eur. Heart J..

[13] Savarese G., Von Haehling S., Butler J., Cleland J.G.F., Ponikowski P., Anker S.D. (2023). Iron Deficiency and Cardiovascular Disease. Eur. Heart J..

[14] Schou M., Bosselmann H., Gaborit F., Iversen K., Goetze J.P., Soletomas G., Rasmussen J., Kistorp C., Kober L., Gustafsson F. (2015). Iron Deficiency: Prevalence and Relation to Cardiovascular Biomarkers in Heart Failure Outpatients. Int. J. Cardiol..

[15] Badura K., Janc J., Wąsik J., Gnitecki S., Skwira S., Młynarska E., Rysz J., Franczyk B. (2024). Anemia of Chronic Kidney Disease—A Narrative Review of Its Pathophysiology, Diagnosis, and Management. Biomedicines.

[16] Van Der Schouw Y.T., Van Der Veeken P.M.W.C., Kok F.J., Koster J.F., Schouten E.G., Hofman A. (1990). Iron Status in the Acute Phase and Six Weeks after Myocardial Infarction. Free Radic. Biol. Med..

[17] Abdelnabi M., Almaghraby A., Benjanuwattra J., Saleh Y., El Azeem A.A., Ghazi R. (2023). The Usefulness of Initial Serum Ferritin Level as a Predictor of In-Hospital Mortality in STEMI. Br. J. Cardiol..

[18] Zeller T., Waldeyer C., Ojeda F., Schnabel R.B., Schäfer S., Altay A., Lackner K.J., Anker S.D., Westermann D., Blankenberg S. (2018). Adverse Outcome Prediction of Iron Deficiency in Patients with Acute Coronary Syndrome. Biomolecules.

[19] Silva C., Martins J., Campos I., Arantes C., Braga C.G., Salomé N., Gaspar A., Azevedo P., Álvares Pereira M., Marques J. (2021). Prognostic Impact of Iron Deficiency in Acute Coronary Syndromes. Rev. Port. Cardiol. Engl. Ed..

[20] Jenča D., Melenovský V., Mrázková J., Šramko M., Kotrč M., Želízko M., Adámková V., Piťha J., Kautzner J., Wohlfahrt P. (2024). Iron Deficiency and All-Cause Mortality after Myocardial Infarction. Eur. J. Intern. Med..

[21] Dziegala M., Kasztura M., Kobak K., Bania J., Banasiak W., Ponikowski P., Jankowska E.A. (2016). Influence of the Availability of Iron during Hypoxia on the Genes Associated with Apoptotic Activity and Local Iron Metabolism in Rat H9C2 Cardiomyocytes and L6G8C5 Skeletal Myocytes. Mol. Med. Rep..

[22] Paterek A., Kępska M., Sochanowicz B., Chajduk E., Kołodziejczyk J., Polkowska-Motrenko H., Kruszewski M., Leszek P., Mackiewicz U., Mączewski M. (2018). Beneficial Effects of Intravenous Iron Therapy in a Rat Model of Heart Failure with Preserved Systemic Iron Status but Depleted Intracellular Cardiac Stores. Sci. Rep..

[23] Florian A., Ludwig A., Rösch S., Yildiz H., Klumpp S., Sechtem U., Yilmaz A. (2014). Positive Effect of Intravenous Iron-Oxide Administration on Left Ventricular Remodelling in Patients with Acute ST-Elevation Myocardial Infarction—A Cardiovascular Magnetic Resonance (CMR) Study. Int. J. Cardiol..

