# Supplementary material for: Iron Deficiency in Acute Coronary Syndrome Treated with Percutaneous Angioplasty—A Factor of Unestablished Significance
Source: Biomedicines. 2026 May 3;14(5):1038. doi: 10.3390/biomedicines14051038 (PMC13203707; doi:10.3390/biomedicines14051038)
Supplement: Supplementary file 1 [file biomedicines-14-01038-s001.zip › biomedicines-4226479-supplementary revised after pub.pdf]

Table S1. Baseline characteristics with the division for ID subtypes. Numbers in second, third, fourth and fifth column represent median (interquartile range Q1-Q3) unless stated otherwise. Presented *P*-value was estimated for difference between three groups – Non-ID, absolute ID and functional ID.

	**Total (n=214)**	**Non-ID (n=114)**	**Absolute ID**	**Functional ID**	***P***
Age, y	69.5 (60.0-75.0)	69.0 (59.0-74.0)	72.0 (63.0-78.0)	69 (59-76)	0.21
Male, n (%)	138 (64.49)	86 (75.4)	8 (26.67)	44 (62.9)	<0.001
BMI, kg/m^2	27.9 (25.0-31.7)	28.41 (25.0-31.4)	26.7 (24.1-30.1)	28 (25.38-32.8)	0.39
Nicotin abuse, n (%)	128 (59.8)	66 (58.0)			
Arterial hypertension, (%)	196 (91.6)	104 (91.2)	28 (93.3)	64 (91.4)	0.95
DM, n (%)	73 (34.1)	43 (29.8)	11 (36.7)	28 (40.0)	0.37
on insulin, n (% of DM patients)	21 (28.8)	9 (22.0)	4 (36.4)	8 (28.6)	0.91
Chronic HF, n (%)	44 (20.6)	26 (22.8)	6 (20.0)	11 (15.7)	0.41
AHF on admission, n (%)	31 (14.5)	14 (12.3)	5 (17.2)	12 (17.1)	0.63
Chronic lung disease, n (%)	32 (15.9)	13 (11.4)	5 (16.7)	13 (18.6)	0.40
Stroke/TIA, n (%)	12 (5.6)	3 (2.6)	3 (10.0)	6 (8.6)	0.13
AF, n (%)	23 (10.8)	11 (9.7)	4 (13.3)	8 (11.4)	0.83
CKD, n (%)	44 (20.6)	22 (19.3)	5 (16.7)	17 (24.3)	0.69
Peptic ulcer disease, n (%)	21 (9.8)	12 (10.5)	2 (6.7)	7 (10.0)	0.79
Anemia, n (%)	43 (20.0)	11 (9.7)	11 (36.7)	21 (30.0)	<0.001
Hb, g/dL	13 (12.9-14.8)	14.2 (13.4-15.2)	13.05 (11.8-14.5)	13.7 (12.5-14.6)	< 0.001
Free iron, µmol/L	12.20 (9.1-15.6)	14 (12.4-17.3)	11.1 (8.3-15.9)	8.4 (6.7-10.1)	< 0.001
Fer, ng/mL	230 (134.0-381.0)	273.0 (165.0-475.0)	62.0 (34.0-87.0)	255.5 (168.0-378.0)	< 0.001
TIBC, µmol/L	56.72 ± 9.2	56.18 ± 8.7	62.32 ± 8.02	55.47 ± 9.86	0.008
TSAT, %	21.00 (16.0-27.0)	27.00 (23.0-31.0)	17.50 (13.00-21.0)	15 (13-17)	< 0.001
Timing of iron parameters sample collection (after ACS diagnosis), days	15 (12.0-18.0)	15 (12.0-18.0)	15 (12-21)	13 (10-18)	0.14
RBC 10^6/mcL	4.56 (4.2-4.9)	4.58 (4.3-4.9)	4.54 (4.15-4.8)	4.55 (4.09-4.9)	0.51
PLT 10^3/mcL	240 (195.0-290.0)	224 (185.0-283.0)	263 (206-293)	252 (197-308)	0.16
sCr, mmol/L	83.40 (69.3-97.5)	83.10 (71.7-95.7)	73.7 (63.6-88.0)	86.3 (69.4-111.4)	0.08
EGFR, ml/min/1.73 m^2	71.3 (56.2-88.7)	74.1 (57.2-92.1)	66.5 (51.0-93.7)	70.45 (52.8-85.3)	0.36

NT-proBNP, pg/mL	621.0 (183.0-1896.0)	516 (162.0-1463.0)	1171 (533-2019)	941.05 (193.0-2534.0)	0.06
hs-cTnT on admission, ng/L	84 (32.0-249.0)	75 (30.0-202.0)	54.5 (30-280)	124.5 (36.0-294.0)	0.10
hs-cTnT peak, ng/L	405 (32.0-249.0)	360.50 (143.5-1935.5)	451.5 (113.0-1199.0)	420 (145-2286)	0.66
**Transthoracic echocardiography**					
LVEF (BiP), %	50.00 (45.0-58.0)	50.00 (45.0-58.0)	55 (50-58)	49.5 (40-56)	0.09
TAPSE, mm	21.78 ± 3.6	21.97 ± 3.6	21.43 ± 3.1	21.63 ± 3.8	0.70
**Invasive coronary angiography and intervention during hospitalization**					
PCI, n (%)	197 (92.1)	102 (89.5)	28 (93.3)	67 (95.7)	0.92
IRA					
LMCA, n (%)	24 (11.2)	10 (8.8)	2 (6.7)	12 (17.1)	0.18
LAD, n (%)	146 (68.2)	79 (69.3)	19 (63.3)	48 (68.6)	0.66
LCx, n (%)	89 (41.6)	52 (45.6)	5 (16.7)	32 (45.7)	0.009
RCA, n (%)	123 (57.5)	60 (52.6)	20 (66.7)	43 (54.6)	0.41
MVD, n (%)	143 (66.8)	79 (69.3)	15 (50.0)	49 (70.0)	0.10
Number of stents implanted					
0, n (%)	17 (7.9)	12 (11.5)	2 (6.7)	3 (4.3)	
1, n (%)	98 (45.8)	49 (43)	19 (63.3)	30 (43.5)	
2, n (%)	64 (29.9)	36 (31.6)	6 (20.0)	22 (31.9)	
3, n (%)	23 (10.8)	12 (10.5)	2 (6.7)	9 (13.0)	
4, n (%)	9 (4.2)	3 (2.6)	1 (3.3)	5 (7.3)	
5, n (%)	3 (1.4)	2 (1.8)	0 (0.0)	1 (1.5)	
Total stents implanted, n	1.00 (1.0-2.0)	1.00 (1.0-2.0)	1 (1.0-2.0)	2 (1.0-2.0)	0.19
Contrast media, ml	110 (80-150)	110 (80-150)	100 (80-120)	120 (100-150)	0.17
EFD, mGy	427.50 (256.0-686.0)	442.00 (278.0-700.0)	250 (141.0-514.5)	440 (314-812)	0.02

AF – atrial fibrillation; AHF – acute heart failure; BiP - biplane; BMI – body-to-mass index; CKD – chronic kidney disease; DM – diabetes mellitus; eGFR – estimated glomerular filtration rate; EFD – effective absorber dose of radiation; Fer – ferritin; Hb – hemoglobin; hs-cTnT – high sensitivity cardiac troponin T; ID – iron deficiency; IRA – infarct related artery; LAD – left anterior descending coronary artery; LCx – left circumflex coronary artery; LMCA – left main coronary artery; LVEF – left ventricular ejection fraction; MVD – multivessel disease; NT-proBNP – N-terminal pro-B-type natriuretic peptide; PCI – percutaneous coronary intervention; PLT – blood platelets; RBC – red blood cells; RCA – right coronary artery; sCr – serum creatinine; TAPSE – tricuspid annular peak systolic excursion; TIA – transient ischemic attack; TIBC – total iron binding capacity; TSAT – transferrin saturation.

Iron status assessment – timing of blood sampling

Median (IQR) time of blood sampling after ACS diagnosis were: 15 (12-18) days for non-ID group, 15 (12-21) days for absolute ID group and 13 (10-18) days for functional ID group and did not differ between each other (*P* for Kruskal-Wallis test 0.14).

To overcome the risk of patient's misclassification and avoid potential association of blood sampling we present results of analyses corrected for sampling time. Based on previously presented calculations we divided patients into two subgroups: early sampling < 15 days after ACS and late

sampling ≥ 15 days after ACS. No differences in FER and TSAT between groups were observed in patients with early sampling and late sampling.

Table S2. Differences in iron parameters in regard to early and late sampling.

	Early sampling	Late sampling	*P* for difference
FER	230 (143-336)	230 (129-455)	0.59
TSAT	20 (14.5-27.5)	22 (17-27)	0.13
Fe	12.1 (8.9-15.8)	12.2 (9.2-15.6)	0.78
TIBC	57.79 ± 10.07	55.83 ± 8.40	0.16

Fe – serum iron concentration; FER – ferritin; TIBC – total iron binding capacity; TSAT – transferrin saturation.

Multivariable linear regression model and risk of multicollinearity

To assess the potential of multicollinearity in multivariable linear regression model we formally assessed collinearity using variance inflation factors (VIFs). All variables demonstrated very low VIF values (TSAT: 1.01, ferritin: 1.16, LVEF: 1.10), indicating negligible multicollinearity.

Table S3. Endpoints breakdown divided into groups of interest – all-cause death and causes of non-elective rehospitalization.

**Group dependent number of endpoints:**				
Endpoint type	No ID	Functional ID	Absolute ID	Total
All-cause death	6	6	0	12
**Non-elective cardiac rehospitalization**				
ACS	7	4	3	14
Angina pectoris	5	4	0	9
Hypertensive emergency	0	1	2	3
Acute HF	3	5	2	10
Pulmonary embolism	1	0	0	1
Arrhytmia	2	1	0	3
Total	18	15	7	**40**

ACS – acute coronary syndrome; HF – heart failure; ID – iron deficiency;

Figure S1. Hazard ratio of ID/ID subtypes in subgroup analysis.

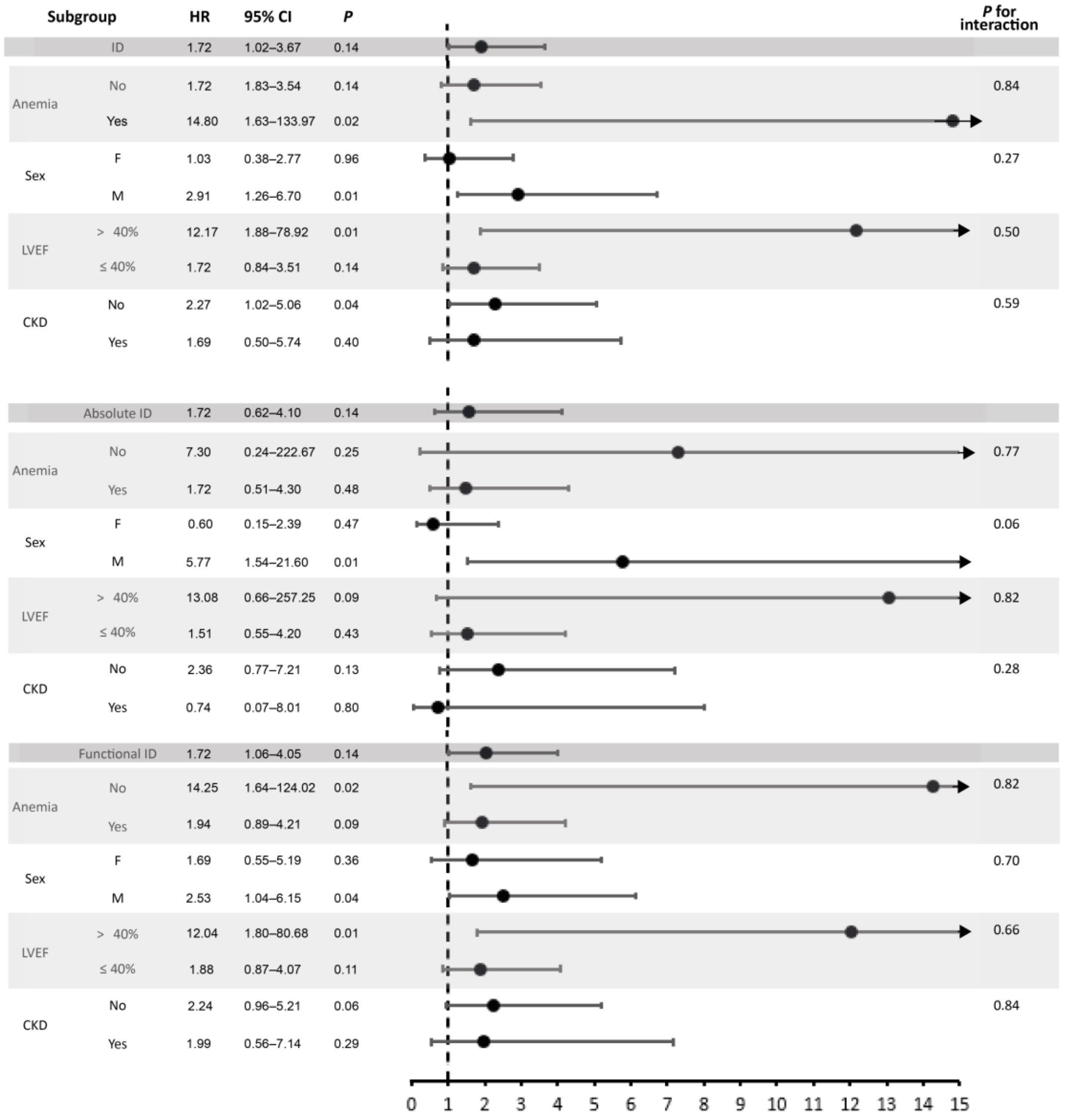

CI – confidence interval; CKD – chronic kidney disease; HR – hazard ratio; LVEF – left ventricular ejection fraction;
